# Supplementary material for: Patterns of Intron Gain and Loss in Fungi
Source: PLoS Biol. 2004 Nov 30;2(12):e422. doi: 10.1371/journal.pbio.0020422 (PMC532390; doi:10.1371/journal.pbio.0020422)
Supplement: Table S1 — Also available at http://genes.mit.edu/NielsenEtAl/. (4.3 MB ZIP). [file pbio.0020422.st001.zip › NielsenEtAl/html/1224.html]

AN6544.1.NCU04188.1.MG03971.1.FG07322.1


```
 CLUSTAL W (1.82) Multiple Sequence Alignments - Introns Inserted


Sequence 1: MG03971.1	171 aa
Sequence 2: FG07322.1	171 aa
Sequence 3: NCU04188.1	203 aa
Sequence 4: AN6544.1	174 aa
Alignment Length: 206 aa
Number Identitical Residues: 96 aa
Alignment Score (without introns) 4690


MG03971.1 	ME--EERLWKFRRPQWLNSVWARNAGVYTSGAL0--------FSLAFYVLLDAAVWSKSP
NCU04188.1	MS--EERLFRFSRPEWLNSAWVRNAGVYAAGAF0SANAMKIQFSLAFYTLLDSAVWSHSP
FG07322.1 	ME--EERLWKFRRPEWLNSVWARNAGVYGAGAI0--------FSIAFYIMLDSAVWSKSP
AN6544.1  	MDGSENRLFRFSKPEWLNNSTVRNAGVYTSGAL0--------FSLGFFFLIDAAAFSHSY
          	*..:*:**::* :*:***.  .****** :**:         **:.*: ::*:*.:*:* 

MG03971.1 	LNPSDTHVTFVDWLPFIFSSLGMLIINSVEKSRLGADSFSYSGSGVAWKARVVLFLGFAC
NCU04188.1	LNASDVHIHFVDWLPFIFSTLGMLIINSVEKARLSSESFSYSGSGVAWKARVVLFLGFAC
FG07322.1 	RNGSDIHVKFVDWLPFIFSSLGMLIINSVEKQRLSADSFSYSGNGVAWKARVVLFLGFAA
AN6544.1  	RNASTVHVKFVDWIPGICSALGMLVINSIEKSRLQADSFSYSGNGVAWKARFVLFLGFAL
          	 * *  *: ****:* * *:****:***:** ** ::******.*******.******* 

MG03971.1 	LAGGMAGGV~CVFVLGYVVPGLGMPQMFMGVENVVANALVGLS2-SAVLWISQNMEDEYS
NCU04188.1	LAGGMAGGV~TVFVLKYVVKDVGFPALRMGVENLVANGLVGLS2-AVVLWISQNMEDEYS
FG07322.1 	LAGGMAGGV~TVFVLKFVVPSVAMPALGMGIENVLSNALVGVS2-SVVLWISQNMEDEYA
AN6544.1  	LAGGLAGSV0TVMVLKYLIKNYPLPTLYFGIANVIANGLVMLS2SSVVLWISQNIEDDYT
          	****:**.*  *:** ::: .  :* : :*: *:::*.** :* ::.*******:**:*:

MG03971.1 	YNLAL~------------~------------
NCU04188.1	YNLAL2STTPSLPPHSKQ0VFMPGTESGNRM
FG07322.1 	YNLSL~------------~------------
AN6544.1  	YNLAL~------------~------------
          	***:*
```
